# Comparison of Lymphocyte Subset Populations in Children From South Africa, US and Europe

**DOI:** 10.3389/fped.2020.00406

**Published:** 2020-07-23

**Authors:** Helen Payne, Denise Lawrie, Martin Nieuwoudt, Mark Fredric Cotton, Diana M. Gibb, Abdel Babiker, Debbie Glencross, Nigel Klein

**Affiliations:** ^1^UCL Great Ormond Street Institute of Child Health, London, United Kingdom; ^2^National Health Laboratory Service, Faculty of Health Science, University of the Witwatersrand, Johannesburg, South Africa; ^3^Institute for Biomedical Engineering (IBE), Stellenbosch University, Stellenbosch, South Africa; ^4^Family Centre for Research With Ubuntu, Stellenbosch University, Cape Town, South Africa; ^5^Clinical Trials Unit, Medical Research Council, London, United Kingdom

**Keywords:** lymphocyte, immunophenotype, reference intervals, intervals, resource-limited, South Africa, pediatric

## Abstract

**Background:** Typically, African healthcare providers use immunological reference intervals adopted from Europe and the United States (US). This may be inappropriate in a setting with many differences including exposure to different environmental stimuli and pathogens. We compared immunological reference intervals for children from Europe and the US with South African children to explore whether healthy children living in settings with high rates of infectious diseases have different baseline immunological parameters.

**Methodology:** Blood was taken from 381 HIV-uninfected children aged between 2 weeks and 13 years of age from a Child Wellness Clinic in an informal settlement in Cape Town to establish local hematological and lymphocyte reference intervals for South African children. Flow-cytometry quantified percentage and absolute counts of the B-cells, NK-cells, and T-cells including activated, naïve, and memory subsets. These parameters were compared to three separate studies of healthy children in Europe and the US.

**Results:** Increased activated T-cells, and natural killer cells were seen in the younger age-groups. The main finding across all age-groups was that the ratio of naïve/memory CD4 and CD8 T-cells reached a 1:1 ratio around the first decade of life in healthy South African children, far earlier than in resource-rich countries, where it occurs around the fourth decade of life.

**Conclusions:** This is the largest data set to date describing healthy children from an African environment. These data have been used to create local reference intervals for South African children. The dramatic decline in the naïve/memory ratio of both CD4 and CD8 T-cells alongside increased activation markers may indicate that South African children are exposed to a wider range of environmental pathogens in early life than in resource-rich countries. These marked differences illustrate that reference intervals should be relevant to the population they serve. The implications for the developing pediatric immune system requires further investigation.

## Introduction

Understanding the immune system in relation to disease pathogenesis and management of infectious diseases is particularly pertinent to children, who are born with an immature immune system that develops rapidly in early life. Understanding what is “normal” in a healthy child should be related to their living environment, and particularly to infectious diseases exposure.

Typically healthcare laboratories, clinical practitioners, and researchers in resource-limited settings use reference intervals adopted from studies that have acquired data from healthy children in resource-rich countries (1–3). These may not be appropriate for assessing health and disease in children from Africa or other distinct environments.

Antiretroviral therapy is now recommended for all patients infected with HIV. Our work has identified that the age and CD4 count at treatment initiation is critical for estimating immune recovery (4), however these CD4 projections are based upon immune reference intervals generated in Europe and USA. Ethnic origin, genetics, climate, altitude, nutrition, and environmental pathogen exposure (5, 6) may influence hematological and immunological parameters, and can vary widely between continents and individual countries. Differences in hematological subsets between resource-rich and resource-limited countries have been reported, including non-genetic neutropenia (7), lower platelet counts (8, 9), and other parameters such as hemoglobin levels, red blood cell counts, haematocrit levels, mean corpuscular volume, and white blood cell counts (10–15).

Differences in lymphocyte parameters, lower CD4 and higher CD8 subsets (12), along with reduced naïve proportions and increased activated CD4 and CD8 T-cells are reported in Ethiopian compared to Dutch adults (16). CD4 percentages are lower in children from Cameroon (17), Kenya (18), Uganda (19), and Malawi (20) compared to European or US reference intervals. There are also differences between African countries in levels of CD8 T-cells, B-cells, and NK cells (21). While local reference intervals in Africa are increasingly being established (11, 12, 17, 18, 22), the range of immunological parameters is limited and statistical comparison across populations has not been explored in detail.

Quantitative differences in immune parameters between children and adults were established relatively recently. For instance, an adult has ~3,000 cells/microlitre of lymphocytes in the peripheral blood, while in children, lymphocyte cell-count/microlitre rises from birth to a maximum of 9,500 between 6 months and one year of age (23, 24), it then follows an exponential decline as the child grows into adulthood. This may be due to several inter-related factors: the progressive involution of the thymus, exposure to antigens, and switch from naïve-to-memory associated with immunological “learning,” change in body size and blood volume associated with growth and the progressive age-related replacement of primary thymic production by peripheral cell division (25). There is currently no agreed standard for the most accurate way to represent these data, however representation of lymphocyte distribution during development is probably best done with mechanistic non-linear modeling rather than using age-categories or empirical methods that represent age as a continuous variable (26).

This study has established how immunological phenotypes change with age in healthy South African children and how this compares with published pediatric data from three resource-rich countries: The Netherlands, Germany and the US (27–29). The potential impact of this work is discussed.

## Materials and Methods

### Participants

Three hundred and eighty-one children aged from 2 weeks to 13 years were recruited from a “Child Wellness Clinic” (CWC) at a community health clinic in an informal settlement of Cape Town, South Africa. The CWC was established primarily as a research clinic, which also aimed to benefit the participants and the wider community for health promotion, education, and screening. Attendance at the CWC was voluntary, and the criteria for recruitment were that the child was well at the time with no chronic medical condition or prescription medications, registered at the health clinic, and attended with their biological mother and hand-held medical record. Maternal HIV-exposure was not excluded. Informed consent was obtained in English or via translator in Afrikaans or Xhosa. The session included clinical history and examination by a pediatrician, plotted anthropometry, assessment of vaccination status (with catch-up as needed), and provision of nutritional supplements and a food voucher. Each participant had phlebotomy of 2–3 mls of blood used for rapid HIV-antibody analysis (Alere Determine®, 4th Generation), full blood count and basic immunophenotyping. HIV-infected children were not included. Stellenbosch University granted ethical approval (M12/01/005) and permission for the study was given by Cape Town Department of Health.

### Laboratory Testing

Blood samples were taken between 9 a.m. and 1 p.m., with 500 μL of the original samples in EDTA couriered at room temperature by air to Johannesburg and processed the following morning at the National Health Laboratory Service, Charlotte Maxeke Johannesburg Academic Hospital (SANAS M0109). Immunophenotypic analysis was performed at the Johannesburg flow cytometry laboratory according to standard operating procedures. Directly labeled antibodies CD3 APC, CD3 FITC, CD16 PE, CD19 FITC, CD45 PerCP, CD45RO PE, CD45RA FITC, HLA Dr APC (Becton Dickinson Immunocytometry Systems (BDIS), San Hose, CA), CD4 FITC, CD8 PE, and CD56 PE (Beckman Coulter, Inc. Miami, Florida) were added in pre-titrated manufacturer optimized concentrations to tubes with 50 μL of well-mixed whole blood. Stained samples were vortexed once and incubated for 30 min. Red blood cells were then lysed using FACS Lysing Solution (BDIS, San Hose, CA). All samples were run on a Becton Dickinson FACSCalibur™ and acquired, and analyzed, using CellQuest™ Pro software. Prior to analysis, basic daily flow cytometer set-up included assessment of Calibrite^TM^ 3 and Calibrite^TM^ APC beads (BDIS, San Hose, CA) to monitor laser, optics, fluidic alignment, linearity, and instrument performance for the FACSCalibur™, according to the manufacturers' standards. Listmode data was stored for retrospective analysis. External CD4 Quality Assessment for CD4 testing was performed through the U.K. NEQAS Immune Monitoring scheme and the NHLS CD4 African Regional External Quality Assessment Scheme (30). Lymphocyte subsets were expressed as a proportion of total lymphocytes determined using bright CD45 expression and side scatter. Specific lymphoid subsets assessed included: total CD3, CD3+/CD4+, CD3+/CD8+, CD3-/CD56+, CD16+/56+, CD3+/HLA DR+, CD3+/CD4+/HLA DR+, CD3+/CD8+/HLA DR+, CD3+/CD4+/45RA+, CD3+/CD4+/45RO+, CD3+/CD8+/45RA+, CD3+/CD8+/45RO+, and CD19+ lymphocytes (Supplement 1A: Gating Strategy to analyse lymphocyte subsets; Supplements 1B,C: Examples of age-related distributions of populations of CD8 and CD4). Absolute cell counts were obtained using a dual platform method; total lymphocyte counts on all samples were obtained on a Beckman Coulter LH750 hematology analyser. All laboratory work and data analysis performed were blinded.

### Studies for Comparison

Three independent studies were used to compare the lymphocyte subsets from our population of healthy South Africa children with those from the US and Europe: Shearer et al. [US (29)], Comans-Bitters et al. [The Netherlands (27)], and Huenecke et al. [Germany (28)]. Shearer et al. and Comans-Bitters et al. used age-categorization (albeit different choices of age-groups), whereas Huenecke et al. presented their data using single exponential regression analysis. We therefore compared these lymphocyte populations according to the presentation of data in each publication. The US and European studies were selected for comparison because the first two are currently being used for reference intervals in South Africa and the latter enabled comparison of the populations using exponential regression techniques. The immunological parameters available for comparison are listed in Table 1.

**Table 1:** Corresponding immunological parameters for comparison between the healthy South African cohort and three published studies: Comans et al. (29), Shearer et al. (28), and Huenecke et al. (30).

**Subset**	**CWC**	**Comans et al**.	**Shearer et al**.	**Huenecke et al**.
No. children	381	358	851	80
Age range	2 wks−13 yrs	1 wk−16 yrs	Birth−18 yrs	2 mths−18 yrs
Country	South Africa	Netherlands	USA	Germany
Total lymphocytes	SSC/CD45 and FSC/SSC	FSC/SSC	FSC/SSC	FSC/SSC
CD3+ T-cells	CD3+	CD3+	CD3+	CD3+
CD4+ T-helper	CD3+CD4+	CD3+CD4+	CD4+	CD3+CD4+
CD8+ Cytotoxic	CD3+CD8+	CD3+CD8+	CD8+	CD3+CD8+
CD4/CD8 ratio	CD3+CD4+ / CD3+CD8+	CD3+CD4+ / CD3+CD8+	NA	CD3+CD4+ / CD3+CD8+
Naïve CD4+	CD4+CD45RA+	NA	CD4+CD45RA+	CD3+CD4+CD45RA+
Memory CD4+	CD4+CD45RO+	NA	CD3+CD4+CD45RO+	CD3+CD4+CD45RO+
Naïve/Memory ratio of CD4+	CD4+CD45RA+ / CD4+CD45RO+	NA	NA	CD3+CD4+CD45RA+/ CD3+CD4+CD45RO+
Naïve CD8+ T-cells	CD8+CD45RA+	NA	CD8+CD45RA+	CD3+CD8+CD45RA+CD28+
Memory CD8+	CD8+CD45RO+	NA	CD3+CD4-CD45RO+	CD3+CD8+CD45RO+
Naïve/Memory ratio of CD8+ T-cells	CD8+CD45RA+ / CD8+CD45RO+	NA	NA	CD3+CD8+CD45RA+CD28+ / CD3+CD8+CD45RO+
Activated T-cells	CD3+HLADR+	CD3+HLADR+	NA	NA
Activated CD4+	CD3+CD4+HLADR+	NA	CD4+HLADR+	CD3+CD4+HLADR+
Activated CD8+	CD3+CD8+HLADR+	NA	CD8+HLADR+	CD3+CD8+HLADR+
CD19+ B-cells	CD19+HLA-DR+	CD19+	CD19+	CD19+
Natural Killer cells	CD3-CD16+CD56+ CD3-CD56+	NA NA	CD16+CD56+ NA	NA CD3-CD56+

*Both cell count per ul and percentage is available for each parameter*.

### Statistical Methods

Subgroups of CWC children with clinical characteristics of interest were compared using Wilcoxon Rank Sum tests to determine whether their lymphocyte subsets differed from the rest of the CWC participants. Comparisons were made between CWC individual age-groups matched to Shearer et al. (29) and Comans-Bitter et al. (27) data (median and 10/90th or 5/95th centiles, respectively) using the Chi-squared goodness-of-fit test, and for differences between the fitted median distributions of each cell marker group using the non-parametric Wilcoxon Rank Sum test.

Comparisons were also made using the results of regression analyses. For this purpose single and double exponential models were fitted to the data. For the single exponential model a similar technique was employed to that used by Huenecke et al. (28), [i.e., the cell counts or percentages of each lymphocyte sub-group were regressed against subject age using a three parameter exponential model: *f* (t) = β_0_ + β_1_ [1 – exp(β_2_ t)], where *t* is age (in weeks) and the betas (B_0, 1, 2, etc_.) are the constants in the equation describing each lymphocyte subset]. Best-fit (median) and 95% CI parameters were determined by minimizing the sum of the square of residuals using MS-Excel's Generalized Reduction Gradient non-linear solver function and a constraint precision of 0.0001. This was also done in R version 3.5.x using packagers nlme and agricolae. To account for the non-normal distribution of the residuals, upper and lower 95% confidence intervals (CIs) were fitted to the data independently.

The double exponential model was formulated from the single exponential model as described above. This model is defined as $f\left(t\right)=\;\frac{\beta_{0}+\beta_{1}\exp\left(-\beta_{2}t\right)}{1+\exp\left(-\beta_{3}t\right)}$, where *t* is the age. The dependent, (i.e., CD marker, variables in this model allows for growth at smaller values of *t*). For larger values of *t*, the denominator 1 + exp (−β_3_*t*) approaches 1 and thus, the model reduces to the single exponential model. The average of β_1_ and β_2_ approximates the counts of the cell-markers at birth. The β_3_ parameter determines the shape of the function. The double exponential model was sufficiently flexible to model either a simple asymptotic reduction, or an initial rise and then fall in CD markers over time, as has been seen in prior mechanistic studies (23, 31).

## Results

**Figure 1:**
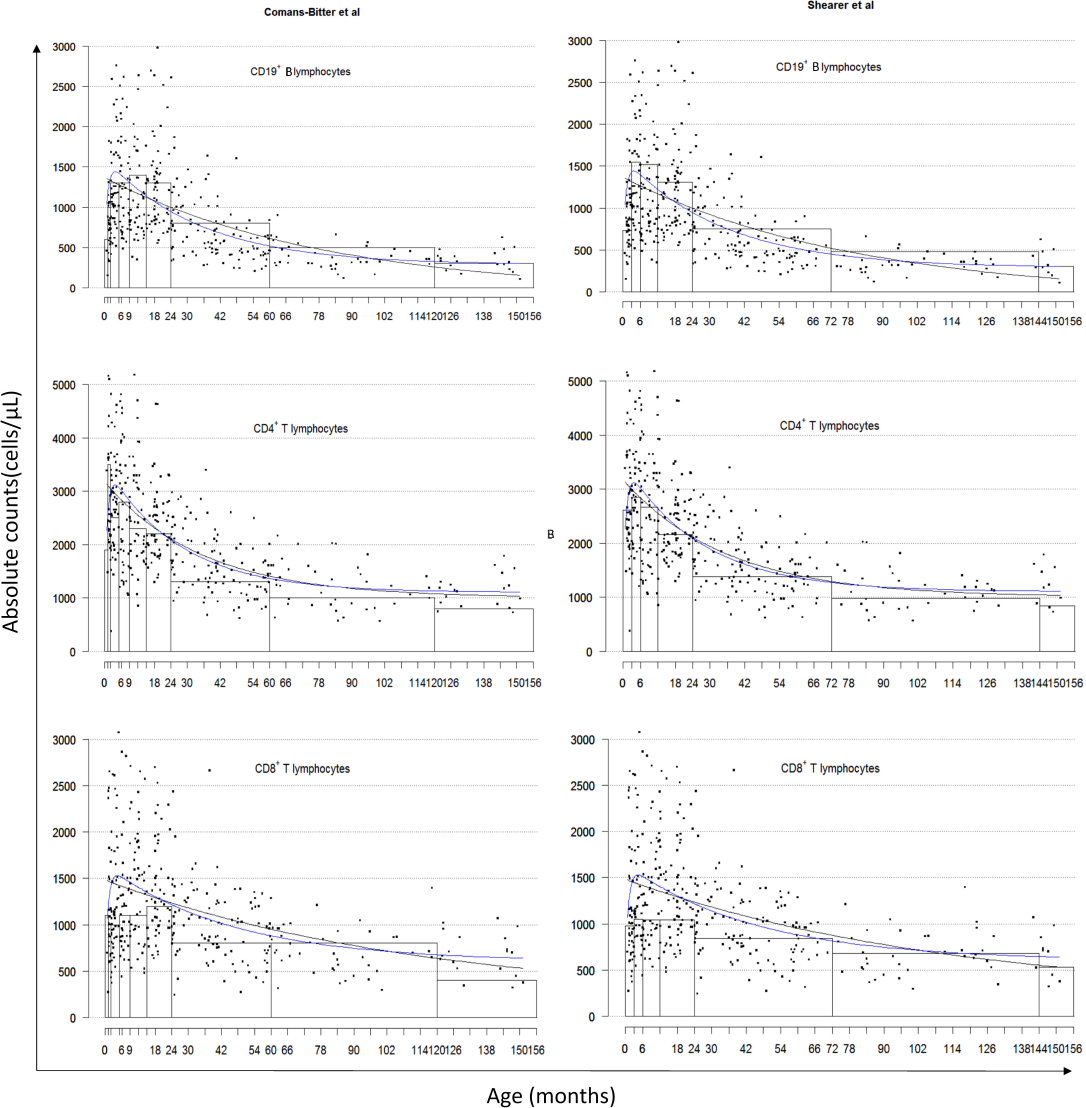
Lymphocyte distributions of South African children by age-category classified by Comans-Bitter et al. (29) left, and Shearer et al. (28) right, with single and double exponential regression lines. Black dots represent individual data points. Black lines represent the single exponential regression curves. Blue lines represent the double exponential regression curves.

All 381 children recruited from the CWC were included in this study and population characteristics are described in Table 2. Lymphocyte distributions of these South African children are represented in Figure 1 using three different presentations of the data: age-category histograms [using the age-categories from Comans-Bitter et al. (29) and Shearer et al. (28)], and single and double exponential regression lines. The double exponential regression lines appear to follow the data and histogram more closely than the single exponential regression.

**Table 2:** Characteristics of the 381 participants from the Child Wellness Clinic (CWC).

	***n* (%) or median [IQR]**
Decimal age at CWC	1.6 [0.6–3.9]
Maternal age at child's birth	25.1 [20.6–32.3]
Ethnicity	Black: 85 (22.3) Mixed ethnic ancestry: 296 (77.7)
Gender	Female: 207 (54.3) Male: 174 (45.7)
Antenatal event (no. & percentage of all CWC recruits)	40 (10.5) *Maternal HIV-infection 14 (3.7), Syphilis 7 (1.9), TB 4 (1), Mental illness 3 (0.8), UTI 2 (0.5), Drug/alcohol abuse 2 (0.5), Growth restriction 2 (0.5), Hepatitis B 2 (0.5), Diabetes 1 (0.3), STD 1 (0.3)*
Neonatal events (Events requiring hospital admission or medication)	57 (14.7) *Prematurity ≤ 36/40: 36 (9.4), ART 7 (1.8), Jaundice 5 (1.3), Suspected infection 3 (0.8), Feeding difficulties 3 (0.8), HIE 1 (0.3); Meningitis 1 (0.3); Pneumonia 1 (0.3)*
Medical history (Past admission, chronic illness or poor growth)	61 (16) *Pneumonia 19 (5), Faltering growth 14 (3.7), TB 11 (2.9), Gastroenteritis 9 (2.4), Operation 2 (0.5), Other 6 (1.6): 1 each of Pyelonephritis, Bacteraemia, Meningitis, PUO, Arthropathy, Skin rash*
Recent illness (>1 wk ago but within past month)	69 (18.1) *URTI 33 (8.7), Skin rash 24 (6.3), Gastroenteritis 10 (2.6), PUO 1 (0.3) UTI 1 (0.3)*
Feeding in first 6 months	Exclusive breast feeding: 231 (60.6) Formula feeding only: 46 (12.1) Mixed breast and formula: 104 (27.3)
Weight-for-age *z*-score	*z*-score 3: 6 (1.6) *z*-score 2: 9 (2.4) *z*-score 1: 45 (11.8) *z*-score 0: 105 (27.6) *z*-score−1: 118 (31) *z*-score−2: 69 (18.1) *z*-score−3: 26 (6.8) *z*-score−4: 3 (0.8)
Immunization status	Fully immunized (records confirm): 322 (84.5) Fully immunized (parental verbal report): 5 (1.3) Immunization not up-to-date: 54 (14.2)

### What Is “Immunologically Normal?”

**Figure 2:**
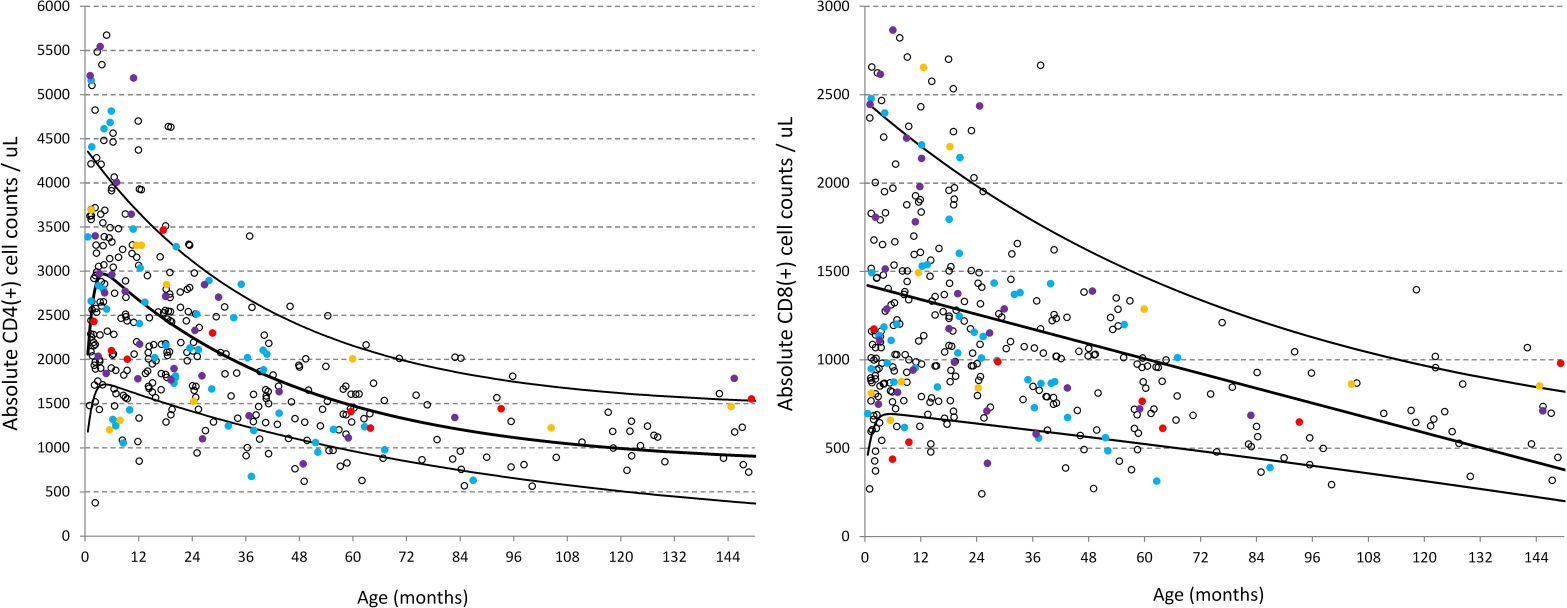
Distribution of CD4 and CD8 (cells/ul) in healthy children from the CWC including clinical subsets of conditions that might be presumed to influence the spread of data. Red circles = past history of a serious childhood illness [e.g., TB, meningitis (*n* = 11); Blue circles = acute recent illness within the past month but more than a week ago e.g., upper respiratory tract infections, gastroenteritis (*n* = 69); Purple circles = maternal conditions during pregnancy e.g., TB, HIV, syphilis (*n* = 28); Yellow circles = prematurity <32 weeks (*n* = 13)]. Black lines represent the best-fit double exponential regression curves with 5 and 95% confidence intervals.

Should children with clinical conditions that could theoretically alter the immunophenotype be included as “normal” for this population? Figure 2 illustrates the distribution of CD4 and CD8 (cells/μL) from the CWC recruits including subgroups of clinical conditions common in this population, that might affect the child's developing immune system and thereby influence the spread of data and reference intervals derived. These conditions include (a) past history of a serious childhood illness [e.g., TB, meningitis (*n* = 11)]; (b) acute recent illness within the past month but more than a week ago [e.g., upper respiratory tract infections, gastroenteritis (*n* = 69)]; (c) maternal infections during pregnancy [e.g., TB, HIV, syphilis (*n* = 28)]; and (d) prematurity <32 weeks (*n* = 13). The exact exponential fit of the regression line for each lymphocyte phenotype examined (as per Table 1) did not appreciably change when these four clinical subsets were in turn removed from the analysis, therefore justifying the inclusion of these children.

“Maternal conditions during pregnancy” was further divided to explore the association of maternal HIV on the lymphocyte subsets (*n* = 14). Differences were detected using the Wilcoxon rank sum test between these 14 children and the rest of the cohort with lower B-cells (CD19+HLADR+, *p* = 0.001) and lower memory CD4 T-cell (CD3+CD4+CD45RO+, *p* < 0.0001) in the HIV-unexposed children. However, removing them from the entire dataset did not affect the exponential regression curves, therefore these children were included as part of this “healthy” population.

### Comparison of Single Exponential Regression Curves of Lymphocyte Populations Between South African and German (28) Children

**Figure 3:**
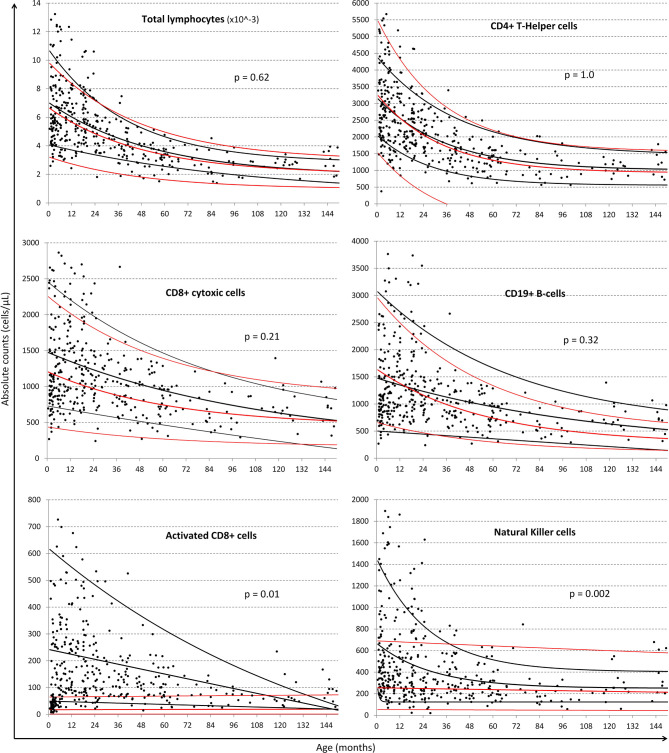
Comparison of regression lines of common lymphocytic markers between South African and German children. Black dots represent individual data-points from the South African cohort. Black lines represent the single exponential regression curves delineated the best-fit, 95 and 5% of the data from the South African cohort. Red lines indicate best-fit, 95 and 5% of the Huenecke's et al. (30) data set. From left to right, top to bottom: absolute lymphocyte count (cells/ul), T-helper cells (CD4+ cells/ul), cytotoxic T-cells (CD8+ cells/ul), B-cells (CD19+ cells/ul), Activated CD8+, and NK cells (CD56+ cells/ul).

Exponential fits were obtained for all lymphocyte subsets listed in Table 1 and a selection are illustrated in Figure 3 using the single exponential fit for purpose of comparison. Absolute cell count curves for the lymphocyte subsets either initially increased or simply descended asymptotically with age. Best-fit, 5 and 95% confidence interval exponential regression curves were fitted for the CWC cohort (black lines) and the Huenecke data from healthy German children for comparison (red lines). A trend toward higher absolute counts of CD8 T-cells and B-cells were seen in South African compared to German children, however significantly higher for NK-cells (*p* = 0.002) and activated CD8 T-cells (*p* = 0.001, Figure 3). Increased CD4 or CD8 activation was associated with decreasing naïve/memory ratio in the South African cohort (cor −0.15, 95% CI [−0.25 −0.05], *p* = 0.003 and cor −0.23, 95% CI [−0.32–0.13], *p* < 0.0001 respectively).

**Figure 4:**
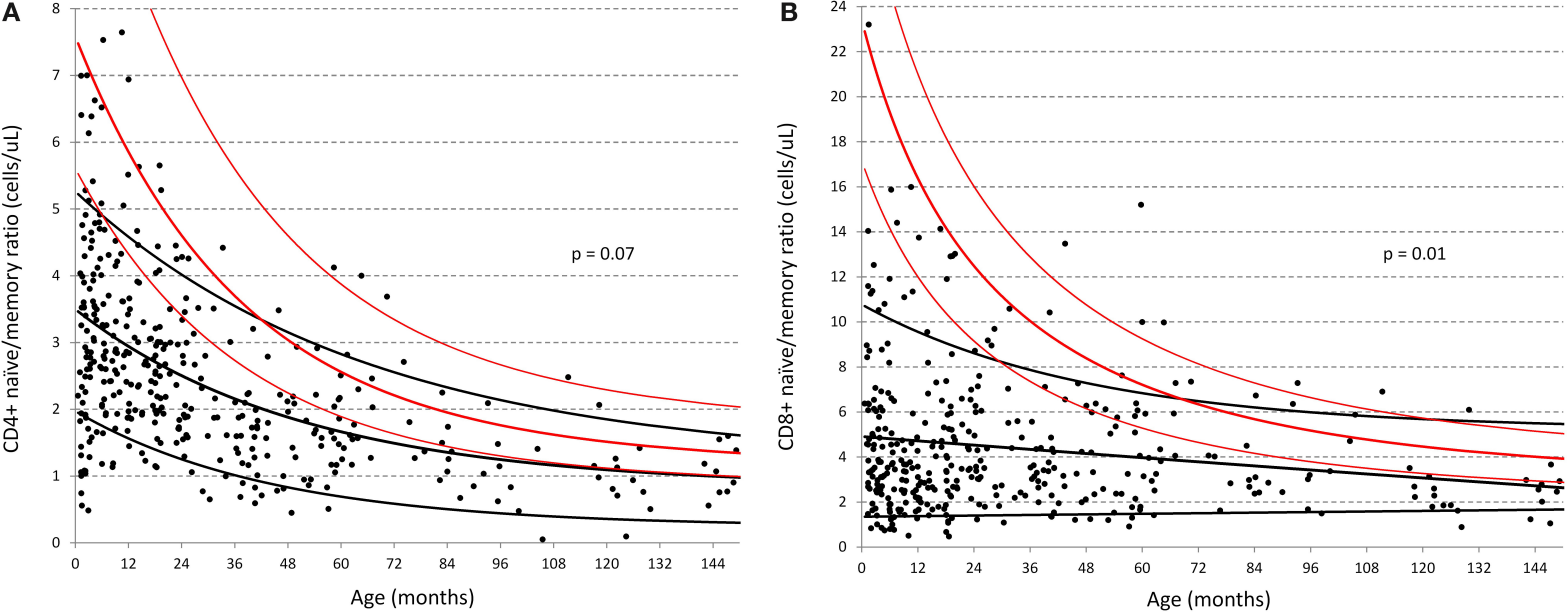
Comparison of naïve:memory ratios of CD4 and CD8 T-cells between South African and German children (30). **(A)** Ratio of naïve/memory CD4+ T-cells in cells/ul; **(B)** Ratio of naïve/memory CD8+ T-cells in cells/ul. Black dots represent individual data-points from the South African cohort. Black lines represent the best-fit single exponential regression curves with 5 and 95% confidence intervals for the South African cohort. Red lines indicate the same but for the data of Huenecke et al. (30).

Marked differences were also seen for both CD4 and CD8 memory populations particularly within the first 3 years of life, as illustrated by the change in naïve/memory ratios with age in Figure 4 (respectively *p* = 0.07 and 0.01 overall). While Huenecke et al.'s data suggests naïve/memory ratios do not reach a 1:1 status (28) until around the third decade of life, it is apparent that this occurs within the first decade in our South African cohort.

### Age-Categorized Data Between South African and US or Dutch Children

Consistent with the above results, the most significant finding when examining the distribution across all age-groups (denoted by the overall *p*-value in Table 3), were the increased proportions of CD4 and CD8 T-cells memory subsets across the entire age range of South African children compared to their US and European counterparts. These children also had lower CD4 and CD8 naïve subsets, particularly at <1 year of age. As illustrated by Figure 4, the data in Table 3 shows that both CD4 and CD8 T-cell naïve/memory ratios differ dramatically at less than a year of age, but not significantly so thereafter.

**Table 3:** Differences in cell marker measurements within specific age-groups between South African children and North- American (Shearer et al.) or Dutch children (Coman et al.).

	**Age groups**								**Overall *p*-value**
**PART A: B-cells**									
**CD19%**	**1 wk-2 mths**	**2-5 mths**	**5-9 mths**	**9-15 mths**	**15-24 mths**	**2-5 yrs**	**5-10 yrs**	**10-16 yrs**	
CWC	13 (8–21.1)	19.1 (9.7–31.4)	19.6 (12.6–38.3)	18.5 (10.6–29.1)	20.1 (10.6–32.6)^*^	15.8 (9.9–30.2)^*^	13 (9–18.4)^*^	11.8 (7.8–16.6)	0.05
Coman	15 (4–26)	25 (14–39)	21 (13–35)	25 (15–39)	28 (17–41)	24 (14–44)	18 (10–31)	16 (8–24)	
**CD19%**	**0–3 mths**	**3–6 mths**	**6–12 mths**		**1–2 yrs**	**2–6 yrs**	**6–12 yrs**	**12+yrs**	
CWC	15.4 (8.8–21.8)	20.1 (12.6–29.6)	18.6 (11.9–27.8)		20 (12.9–30)	15.7 (11–24.1)	11.9 (9.4–16.7)^*^	11.5 (8.6–13.2)	0.16
Shearer	15 (6–32)	2 (11–41)	24 (14–33)		21 (14–33)	21 (14–33)	18 (13–27)	14 (6–23)	
**PART B: Activation markers and cytotoxic T–cells**									
**CD8%**	**1 wk−2 mths**	**2–5 mths**	**5–9 mths**	**9–15 mths**	**15–24 mths**	**2–5 yrs**	**5–10 yrs**	**10–16 yrs**	
CWC	18.1 (10.7–28.9)	19.4 (11.8–28.6)	22.5 (12.1–31.7)	24.3 (13.3–34.6)	24.2 (13.8–34.9)	24.4 (15.7–35.6)	25.8 (15.1–34.8)	23.4 (18–30.8)^**^	0.13
Coman	16 (9–23)	17 (11–25)	18 (13–26)	18 (12–28)	20 (11–32)	24 (14–33)	28 (19–34)	23 (9–35)	
**CD3+HLA–DR+%**									
CWC	4.7 (2.6–12.1)^***^	6 (2.6–20.1)^***^	8.5 (3.2–19.3)^***^	9.5 (3.7–19.2)^***^	9 (2.9–18.5)	8.4 (4.1–19.9)	6.9 (4.1–19.9)	7.5 (4–10.5)^**^	0.01^**^
Coman	5 (1–38)	3 (1–9)	3 (1–7)	4 (2–8)	6 (3–12)	6 (3–13)	7 (3–14)	4 (1–8)	
**CD4+HLA–DR+%**	**0–3 mths**	**3–6 mths**	**6–12 mths**		**1–2 yrs**	**2–6 yrs**	**6–12 yrs**	**12+yrs**	
CWC	4.0 (2.4–6.0)	4.2 (1.9–8.2)	4.3 (2.5–7.9)		5.6 (2.9–9.6)	5.7 (3.0–8.6)	4.3 (2.3–8.7)	4.2 (2.5–6.6)	0.09
Shearer	3 (2–6)	5 (2–10)	5 (2–11)		6 (2–11)	7 (3–12)	6 (3–13)	7 (4–11)	
**CD8+HLA–DR+%**									
CWC	4.7 (2–20.6)	9.6 (2.5–31.7)^***^	16.9 (6.6–30.2)		14.4 (5.5–28.5)	12.5 (6–23.5)	8.8 (5.5–21.4)	11.7 (7.9–15.6)	1.0
Shearer	5 (2–20)	7 (3–17)	10 (4–27)		16 (6–33)	16 (7–37)	12 (6–29)	12 (5–25)	
**PART C: Naïve and memory subsets**									
**CD4+CD45RA+%**	**0–3 mths**	**3–6 mths**	**6–12 mths**		**1–2 yrs**	**2–6 yrs**	**6–12 yrs**	**12+yrs**	
CWC	72.9 (51–83.8)^*^	78.4 (62.4–84.4)	73.9 (63.8–81.7)		72 (62.6–81.6)	63.8 (47–76.4)	54.7 (40.2–67.8)	48.5 (34.5–60)	0.25
Shearer	90 (64–95)	90 (77–94)	86 (64–93)		81 (63–91)	71 (53–86)	59 (46–77)	53 (33–66)	
**CD4+CD45RO+%**									
CWC	27 (16.2–48.9)^***^	22 (15.6–37.6)^***^	26 (18.3–36.2)^***^		28 (18.4–37.4)^***^	36.2 (23.6–53)^***^	45.3 (32.2–60)^***^	51.5 (40–65.5)^***^	0.01^**^
Shearer	10 (2–22)	8 (3–16)	9 (5–18)		12 (7–20)	16 (9–26)	21 (13–30)	28 (18–38)	
**CD4+CD45RO+cells/ul** **×** **10**^**3**^									
CWC	0.85(0.32–1.69)	0.64(0.39–1.29)	0.58(0.39–1.07)		0.63(0.40–1.03)	0.56(0.38–0.78)	0.48(0.32–0.74)	0.64(0.40–0.73)	0.0006
Shearer	0.32 (0.06–0.90)	0.33 (0.12–0.63)	0.34 (0.16–0.80)		0.40 (0.21–0.85)	0.36 (0.22–0.66)	0.35 (0.23–0.63)	0.38 (0.24–0.70)	^***^
**Naïve/memory CD4 ratio cells/ul**									
CWC	2.7 (1–5.2)^***^	3.6 (1.7–5.4)^*^	2.8 (1.8–4.5)		2.6 (1.7–4.4)	1.8 (0.9–3.2)	1.2 (0.7–2.1)	0.9 (0.5–1.5)	0.18
Shearer	7.1 (4.1–20)	7 (5.9–10.8)	6.5 (4.6–6.9)		4.1 (3.4–4.8)	2.7 (2.3–2)	1.6 (1.6–1.4)	1.1 (1.1–1)	
**CD8+CD45RA+%**									
CWC	83.2 (65.9–92)	76.7 (63–89.8)^**^	77.1 (56.3–86.8)^*^		77.4 (62.6–89.3)	78.1 (62–88.2)	75.4 (64.2–87.1)	66.9 (52.2–77.1)	0.005^**^
Shearer	93 (80–99)	94 (85–98)	91 (75–97)		89 (71–98)	86 (69–97)	80 (63–92)	79 (61–91)	
**CD8+CD45RO+%**									
CWC	16.8 (8–34.1)^***^	23.3 (10–37.2)^***^	23 (13.2–43.7)^***^		22.6 (11–37.4)^***^	21.9 (11.8–38)^***^	24.6 (13–35.8)^***^	33.1 (23–47.8)^***^	0.002^**^
Shearer	3 (1–9)	3 (1–7)	3 (1–8)		6 (2–12)	9 (4–16)	12 (4–21)	13 (4–23)	
**CD8+CD45RO+** **cells/ul** **×** **10**^**3**^								
CWC	0.14(0.06–0.63)	0.26(0.09–0.66)	0.34(0.13–0.66)		0.29(0.11–0.69)	0.19(0.09–0.39)	0.15(0.08–0.24)	0.23(0.09–0.31)	0.087
Shearer	0.10 (0.03–0.33)	0.12 (0.03–0.29)	0.12 (0.04–0.33)		0.23 (0.06–0.57)	0.19 (0.09–0.44)	0.21 (0.07–0.39)	0.16 (0.06–0.31)	
**Naïve/memory CD8 ratio cells/ul**									
CWC	5 (1.9–11.5)	3.3 (1.7–8.8)^**^	3.4 (1.3–6.6)^**^		3.4 (1.7–8.3)	3.6 (1.6–7.4)	3.1 (1.8–6.8)	2 (1.1–3.4)	0.25
Shearer	8.7 (4.5–15)	7.6 (4.8–18.3)	7.3 (4.5–12)		4.1 (3–8.2)	3.5 (2.5–4.2)	2.6 (2.3–4.4)	2.5 (2.3–4)	
**Numbers**	1 wk−2 mths	2–5 mths	5–9 mths	9–15 mths	15–24 mths	2–5 yrs	5–10 yrs	10–16 yrs	
CWC, n	13	46	105	70	33	33	35	23	
Coman, n	22	53	38	46	59	104	40	19	
**Numbers**	0–3 mths	3–6 mths	6–12 mths		1–2 yrs	2–6 yrs	6–12 yrs	12–18 yrs	
CWC, *n*	45	40	54		79	118	37	8	
Shearer, *n*	~90	~90	~90		~90	~90	~90	~90	

*(A) Differences seen in B-cells; (B) Activation markers and cytotoxic T-cells; (C) Naïve-memory subsets. Information is displayed within the context of the reference ranges of each data set. Measured data from the Child Wellness Clinic (CWC) has been organized to match the age groups of the comparison studies. Reference ranges from Shearer et al. (28) are medians with 10-90th centiles, and for Comans-Bitter et al. (29) are medians with 5-95th centiles. Categories with significant differences are highlighted in red. Age-category comparisons were performed using the CHI-squared test*,

* *indicates significance p < 0.05*,

** *p < 0.01*,

*** *p < 0.001*.

*Shearer et al. included 807 healthy children up until 18 years, however, explicit numbers per age group were not provided in their publication, although each category included ~90 children except 3–6 months (81–89). The ‘overall p-value' is the non-parametric Wilcoxon Rank Sum test for differences in the rank order of the median distributions*.

Table 3 illustrates a significantly higher CD3 HLA-DR+ % in South African children aged 1 week to 15 months, and 10–16 years compared to children from the Netherlands; and significantly higher CD8 HLA-DR+% in South African children aged 2–5 months compared to children from the US. Additional differences were identified between lymphocyte subset distributions within individual age-categories in South African children vs. those from the US and The Netherlands including: lower B-cells in children aged from 15 months to 13 years; and higher percentage of CD8 T-cells in 10–16 years olds.

## Discussion

The dearth of local pediatric reference range data in South Africa (32) prompted this study to establish a relevant local set laboratory reference values to ensure that health care, treatment, and monitoring is appropriate for the population of children being cared-for (33). The data we have generated may be representative of lymphocyte subsets in children living in resource-limited communities who are more likely to be exposed to significant diseases, such as TB and HIV, than their counterparts in resource-rich countries.

Our main finding was the dramatic difference in naïve/memory ratios of T-cell populations in South African compared to US and European children. Parity between these populations of cells was reached some three decades earlier than observed in the German population (30). This has been noted before (31, 34), and could be explained by a reduction in thymic output with depletion of the naïve T-cell pool and/or accompanied by expansion of memory cells as naïve T-cells encounter antigen and memory populations proliferate (35). Until now, characterization of this transition throughout the first decade of life has not been described, nor compared across continents where genetics, nutrition, and environmental antigenic exposure differ extensively.

This increased rate of decline with age in naïve/memory ratio of T-cell populations seems most likely to be due to the induction of immune-activation by increased exposure to environmental pathogens as seen in the South African study population. This is reflected by the increased proportions of CD8 T-cells, natural killer cells and activated T-cells were demonstrated in healthy South African children compared to their US or European counterparts, and increased CD4 or CD8 activation was indeed associated with decreasing naïve/memory ratio. Environmental exposure to common pathogens such as herpesvirus, cytomegalovirus, and Epstein-Barr virus may drive the switch from naïve to memory T-cells; however exposure *per se* may not be the only factor and the abundance of environmental pathogens (36), poor nutritional status and high levels of microbial translocation (37) may also drive the immune response to such pathogens (38, 39). Background immune-activation may be particularly relevant in the current climate of the COVID19 pandemic, whereby pre-existing immune activation might predispose the individual to a more inflammatory response to a new pathogen than a child with an unactivated immune system (40).

It is not possible in our study to determine whether differences seen are related to environmental exposure or genetics. The European studies for comparison do not describe ethnicity, and although the US study reports the majority of their cohort to be of African-American race (58%), the genetics are likely to differ from an African population. While our cohort broadly represents the general population of South Africa in terms of socio-economic background, it does not represent ~25% of the South African population that are relatively wealthier with well-equipped and sanitized home and school environments (41), and thereby might have comparatively less disease exposure and potentially different “normal” immunological phenotypes compared to the participants of the CWC.

The inclusion of the children with histories of significant illnesses, maternal infections during pregnancy, recent illnesses, and prematurity <32 weeks gestational age in the CWC healthy cohort might be a source of debate. However, since the prevalence in the CWC cohort is similar to the study population and there is no clear biological evidence to implicate the effect of these conditions on the child's developing immune system we considered it acceptable to include these conditions. The sub-analyses performed on the 14 children who were born to HIV-infected mothers did not affect the overall regression curves in this study due to the small number and age distribution of the group. A larger study is warranted to explore these potential differences in more detail, especially since only 3.7% of children in our cohort were HIV-exposed compared to recent estimates of 30% of infants born in the public sector in South Africa (42, 43). This low rate of HIV-exposure might be explained by the fact that the clinic was promoted as a “healthy child” clinic where a HIV test would be done on all children, and this may have deterred HIV-infected mothers.

There are limitations to the outcomes of the comparison of lymphocyte subsets between South African children and the three other studies in children from the US and Europe. These three cohorts come from contrasting environments, and the data is generated from studies that used non-identical methodologies. There are multiple practical factors that might influence data derived from flow cytometric studies including sample transport, storage, and preparation, choice of fluorochrome-conjugated antibodies and immunological markers to define subsets of interest. These factors make direct comparison between such studies challenging. In an attempt to minimize the effect of these factors, exponential regression curves were used to compare the changes in immunological parameters across the age-range examined and this approach should help to reduce the influence of confounding factors.

When multiple statistical comparisons are performed, as done in Table 3, there is potential that significant differences detected may be due to chance rather than biological plausibility. These calculations were not adjusted for multiple comparisons because the covariate data from the other studies was not available, and adjustment would have been unlikely to add additional information of value (44). A combination of statistical approaches have been applied to the analysis of these data-sets, however regardless of the statistical approach employed, the main findings of rapid and early transition of naïve CD4 and CD8 T-cells to their respective memory populations in the CWC, was concordant across all three of the compared international studies. Although our study has several limitations, it provides a starting point for exploring differences in immunological phenotypes and the optimal way to characterize the lymphocyte distributions as they change with age. We have illustrated a double exponential model that account for the possibility that cell counts may increase and peak during the 1st year of life with subsequent decline.

The purpose of the CWC was to collect immunological data from healthy South African children to establish local reference intervals (33), since in South Africa clinicians and laboratories had been using a combination of the reference intervals published by Comans-Bitter et al. and Shearer et al. from the US and Europe. A number of important differences between the CWC and these international studies were found, highlighting the value of having contextually appropriate reference intervals available. Although no gross difference was identified in the numbers of lymphocyte subsets most commonly used in clinical practice such as CD4, CD8, and CD4/CD8 ratios, a dramatic and significant difference was demonstrated in the rapid early decline of the naïve/memory ratios of both CD4 and CD8 T-cells alongside increased lymphocyte activation in this pediatric population. While providing valuable insight into the developing pediatric immune system within an African context, the long-term health implications of these findings require further investigation.

## Data Availability Statement

The datasets generated for this study are available on request to the corresponding author.

## Ethics Statement

The studies involving human participants were reviewed and approved by Stellenbosch University granted ethical approval (M12/01/005) and permission for the study was given by Cape Town Department of Health. Written informed consent to participate in this study was provided by the participants' legal guardian/next of kin.

## Author Contributions

HP and DG conceived the study. HP organized, conducted the CWC, and prepared the manuscript. DL processed and analyzed the specimens. MN and HP performed the statistical analysis. NK, DMG, AB, MC, DG, and Robin Callard, contributed to the study design and data interpretation. All authors have contributed to the writing of the manuscript and approved the final draft for submission.

## Conflict of Interest

The authors declare that the research was conducted in the absence of any commercial or financial relationships that could be construed as a potential conflict of interest.

## References

[1] Greer JP Foerster J Rodgers GM Paraskevas F Glader B Arber DA Wintrobe's Clinical Hematology. 14th ed Philadelphia, PA: Lippincott Williams and Wilkins (2018).

[2] Karita E Ketter N Price MA Kayitenkore K Kaleebu P Nanvubya A. CLSI-derived hematology and biochemistry reference intervals for healthy adults in Eastern and southern Africa. PLoS ONE. (2009) 4:e4401. 10.1371/journal.pone.0004401 19197365 PMC2632744

[3] Tugume SB Piwowar ME Lutalo T Mugyenyi NP Grant MR Mangeni WF. Hematological reference ranges among healthy Ugandans. Clin Diagn Lab Immunol. (1995) 2:233–5. 10.1128/CDLI.2.2.233-235.1995 7697535 PMC170134

[4] Picat MQ Lewis J Musiime V Prendergast A Nathoo K Kekitiinwa A. Predicting patterns of long-term CD4 reconstitution in HIV-infected children starting antiretroviral therapy in sub-Saharan Africa: a cohort-based modelling study. PLoS Med. (2013) 10:e1001542. 10.1371/journal.pmed.1001542 24204216 PMC3812080

[5] European Collaborative Study. Are there gender and race differences in cellular immunity patterns over age in infected and uninfected children born to HIV-infected women? J Acquir Immune Defic Syndr. (2003) 33:635–41. 10.1097/00126334-200308150-00013 12902809

[6] Okebe J Mwesigwa J Agbla SC Sanya-Isijola F Abubakar I D'Alessandro U. Seasonal variation in haematological and biochemical reference values for healthy young children in The Gambia. BMC Pediatrics. (2016) 16:5. 10.1186/s12887-016-0545-6 26754650 PMC4710011

[7] Thobakgale CF Ndung'u T. Neutrophil counts in persons of African origin. Curr Opin Hematol. (2014) 21:50–7. 10.1097/MOH.0000000000000007 24257098

[8] Dosoo DK Kayan K Adu-Gyasi D Kwara E Ocran J Osei-Kwakye K. Haematological and biochemical reference values for healthy adults in the middle belt of Ghana. PLoS ONE. (2012) 7:e36308. 10.1371/journal.pone.0036308 22558429 PMC3338654

[9] Haileamlak A Muluneh AT Alemseged F Tessema F Woldemichael K Asefa M. Hematoimmunological profile at gilgel gibe field research center, Southwest ethiopia. Ethiop J Health Sci. (2012) 22:39–50. 23319839 PMC3542742

[10] Menard D Mandeng M Tothy MB Kelembho EK Gresenguet G Talarmin A. Immunohematological reference ranges for adults from the Central African Republic. Clin Diagn Lab Immunol. (2003) 10:443–5. 10.1128/CDLI.10.3.443-445.2003 12738646 PMC154963

[11] Lugada ES Mermin J Kaharuza F Ulvestad E Were W Langeland N. Population-based hematologic and immunologic reference values for a healthy Ugandan population. Clin Diagn Lab Immunol. (2004) 11:29–34. 10.1128/CDLI.11.1.29-34.2004 14715541 PMC321349

[12] Lawrie D Coetzee LM Becker P Mahlangu J Stevens W Glencross DK. Local reference ranges for full blood count and CD4 lymphocyte count testing. South Afr Med J. (2009) 99:243–8. 19588777

[13] Lawrie D Coetzee LM Glencross DK. Iron deficiency anaemia in healthy South African women despite iron fortification. South Afr Med J. (2008) 98:606–7. 18928037

[14] Eller LA Eller MA Ouma B Kataaha P Kyabaggu D Tumusiime R. Reference intervals in healthy adult Ugandan blood donors and their impact on conducting international vaccine trials. PLoS ONE. (2008) 3:e3919. 10.1371/journal.pone.0003919 19079547 PMC2593783

[15] Adetifa IM Hill PC Jeffries DJ Jackson-Sillah D Ibanga HB Bah G. Haematological values from a Gambian cohort–possible reference range for a West African population. Int J Lab Hematol. (2009) 31:615–22. 10.1111/j.1751-553X.2008.01087.x 18631172

[16] Messele T Abdulkadir M Fontanet AL Petros B Hamann D Koot M. Reduced naive and increased activated CD4 and CD8 cells in healthy adult Ethiopians compared with their Dutch counterparts. Clin Exp Immunol. (1999) 115:443–50. 10.1046/j.1365-2249.1999.00815.x 10193416 PMC1905237

[17] Sagnia B Ateba Ndongo F Ndiang Moyo Tetang S Ndongo Torimiro J Cairo C Domkam I. Reference values of lymphocyte subsets in healthy, HIV-negative children in Cameroon. Clin Vaccine Immunol. (2011) 18:790–5. 10.1128/CVI.00483-10 21411603 PMC3122514

[18] Embree J Bwayo J Nagelkerke N Njenga S Nyange P Ndinya-Achola J. Lymphocyte subsets in human immunodeficiency virus type 1-infected and uninfected children in Nairobi. Pediatr Infect Dis J. (2001) 20:397–403. 10.1097/00006454-200104000-00006 11332664

[19] Bunders M Lugada E Mermin J Downing R Were W Thorne C Within and between race differences in lymphocyte, CD4+, CD8+ and neutrophil levels in HIV-uninfected children with or without HIV exposure in Europe and Uganda. Ann Trop Paediatr. (2006) 26:169–79. 10.1179/146532806X120255 16925953

[20] Mandala WL MacLennan JM Gondwe EN Ward SA Molyneux ME MacLennan CA. Lymphocyte subsets in healthy Malawians: implications for immunologic assessment of HIV infection in Africa. J Allergy Clin Immunol. (2010) 125:203–8. 10.1016/j.jaci.2009.10.010 19944455 PMC2887487

[21] Saathoff E Schneider P Kleinfeldt V Geis S Haule D Maboko L. Laboratory reference values for healthy adults from southern Tanzania. Trop Med Int Health. (2008) 13:612–25. 10.1111/j.1365-3156.2008.02047.x 18331386

[22] Buchanan AM Muro FJ Gratz J Crump JA Musyoka AM Sichangi MW. Establishment of haematological and immunological reference values for healthy Tanzanian children in Kilimanjaro Region. Trop Med Int Health. (2010) 15:1011–21. 10.1111/j.1365-3156.2010.02585.x 20636301 PMC3024440

[23] Bains I Antia R Callard R Yates A. Quantifying the development of the peripheral naive CD4 T cell pool in humans. Blood. (2009) 113:5480–7. 10.1182/blood-2008-10-184184 19179300 PMC2689049

[24] Idigbe EO Audu R Iroha EO Akinsulie AO Temiye EO Ezeaka VC. T-lymphocyte subsets in apparently healthy nigerian children. Int J Pediatr. (2010) 2010:474380. 10.1155/2010/474380 20169116 PMC2821635

[25] de Boer RJ Perelson AS. quantifying T lymphocyte turnover. J Theor Biol. (2013) 327:45–87. 10.1016/j.jtbi.2012.12.025 23313150 PMC3640348

[26] Wade AM des AE. Age-related reference ranges: significance tests for models and confidence intervals for centiles. Stat Med. (1994) 13:359–67. 10.1002/sim.4780132207 7855469

[27] Comans-Bitter WM de Groot R van den Beemd R Neijens HJ Hop WC Groeneveld K. Immunophenotyping of blood lymphocytes in childhood. Reference values for lymphocyte subpopulations. J Pediatrics. (1997) 130:388–93. 10.1016/S0022-3476(97)70200-2 9063413

[28] Huenecke S Behl M Fadler C Zimmermann SY Bochennek K Tramsen L. Age-matched lymphocyte subpopulation reference values in childhood and adolescence: application of exponential regression analysis. Eur J Haematol. (2008) 80:532–9. 10.1111/j.1600-0609.2008.01052.x 18284628

[29] Shearer WT Rosenblatt H Gelman RS Oyomopito R Plaeger S Stiehm ER. Lymphocyte subsets in healthy children from birth through 18 years of age: the Pediatric AIDS Clinical Trials Group P1009 study. J Allergy Clin Immunol. (2003) 112:973–80. 10.1016/j.jaci.2003.07.003 14610491

[30] Glencross DK Aggett H Stevens WS Mandy F. African regional external quality assessment for CD4 T-cell enumeration: development, outcomes, and performance of laboratories. Cytometry B Clin Cytom. (2008) 74(Suppl.1):S69–79. 10.1002/cyto.b.20397 18228560

[31] Tsegaye A Wolday D Otto S Petros B Assefa T Alebachew T. Immunophenotyping of blood lymphocytes at birth, during childhood, and during adulthood in HIV-1-uninfected Ethiopians. Clin Immunol. (2003) 109:338–46. 10.1016/j.clim.2003.08.008 14697749

[32] Kiepiela P Coovadia HM Coward P Woodhead R Abdool-Karim SS Becker P. Age-related lymphocyte sub-population changes among healthy Africans from birth to adulthood. Ann Trop Paediatr. (1989) 9:199–205. 10.1080/02724936.1989.11748633 2482000

[33] Lawrie D Payne H Nieuwoudt M Glencross DK. Observed full blood count and lymphocyte subset values in a cohort of clinically healthy South African children from a semi-informal settlement in Cape Town. S Afr Med J. (2015) 105:589–95. 10.7196/SAMJnew.7914 26428758

[34] Provinciali M Moresi R Donnini A Lisa RM Reference values for CD4+ and CD8+ T lymphocytes with naive or memory phenotype and their association with mortality in the elderly. Gerontology. (2009) 55:314–21. 10.1159/000199451 19190395

[35] Bains I Thiebaut R Yates AJ Callard R. Quantifying thymic export: combining models of naïve T cell proliferation and TCR excision circle dynamics gives an explicit measure of thymic output. J Immunol. (2009) 183:4329–36. 10.4049/jimmunol.0900743 19734223

[36] Ngonghala C Pluckinski MM Murray MB Farmer PE Barrett CB Keenan DC. Poverty, disease, and the ecology of complex systems. PLoS Biol. (2014) 12:e1001827. 10.1371/journal.pbio.1001827 24690902 PMC3972083

[37] Prendergast A Kelly P. Enteropathies in the developing world: neglected effects on global health. Am J Trop Med Hygiene. (2012) 86:756–63. 10.4269/ajtmh.2012.11-0743 22556071 PMC3335677

[38] Hickman D Jones MK Zhu S Kirkpatrick E Ostrov DA Wang X. The effect of malnutrition on norovirus infection. mBio. (2014) 5:e01032–13. 10.1128/mBio.01032-13 24595373 PMC3958801

[39] Kugelberg E. Innate lymphoid cells: nutrients direct immune balance. Nat Rev Immunol. (2014) 14:137. 10.1038/nri3626 24503524

[40] Vabret N Britton GJ Gruber C Hegde S Kim J Kuksin M. Immunology of COVID-19: current state of the science. Immunity. (2020) 52:910–41. 10.1016/j.immuni.2020.05.002 32505227 PMC7200337

[41] Statistics South Africa Statistics South Africa, Census 2011. Pretoria (2011).

[42] Medical Research Council South African School of Public Health University of the Western Cape; National Department of Health South Africa: Centers for Disease Control Prevention/PEPFAR; National Institute for Communicable Diseases/National Health Laboratory Service; Wits Paediatrics HIV Diagnostics; UNICEF. Evaluation of the Effectiveness of the National Prevention of Mother-to-Child Transmission (PMTCT) Programme on Infant HIV Measured at Six Weeks Postpartum in South Africa. Pretoria: PEPFAR/ US Centers for Disease Control & Prevention (2010).

[43] Department of Health South Africa National Antenatal Sentinel HIV & Syphillis Survey Report. Pretoria: Department of Health South Africa (2017).

[44] Gelman A Hill J Yajima M Why we (Usually) don't have to worry about multiple comparisons. J Res Educ Effectiveness. (2012) 5:189–211. 10.1080/19345747.2011.618213

