# Supplementary figures and images for: Comparison of Lymphocyte Subset Populations in Children From South Africa, US and Europe
Source: Front Pediatr. 2020 Jul 23;8:406. doi: 10.3389/fped.2020.00406 (PMC7390891; doi:10.3389/fped.2020.00406)

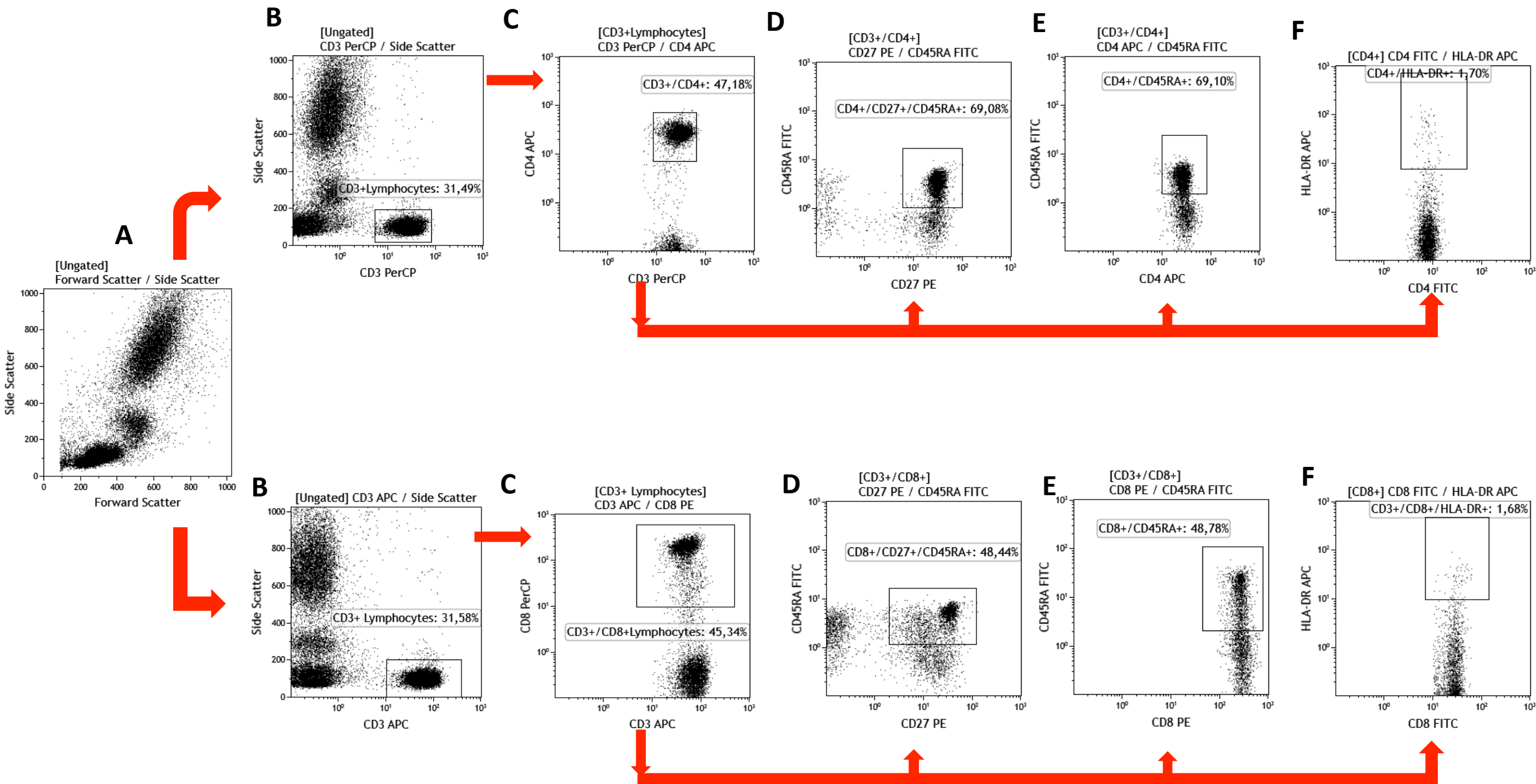

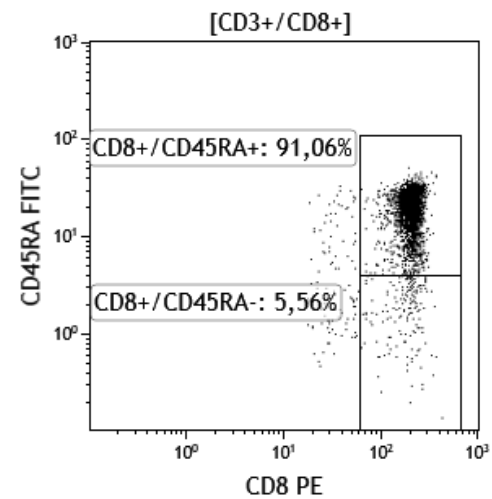

3- 12 weeks

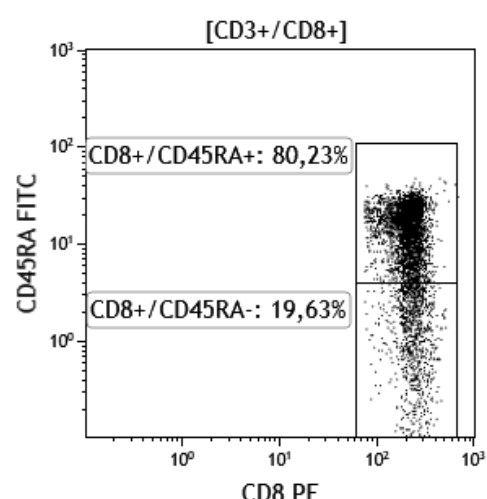

12 - 24 weeks

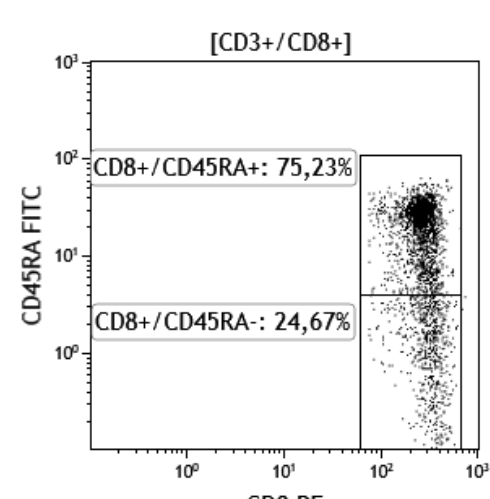

24-48 weeks

427weeks

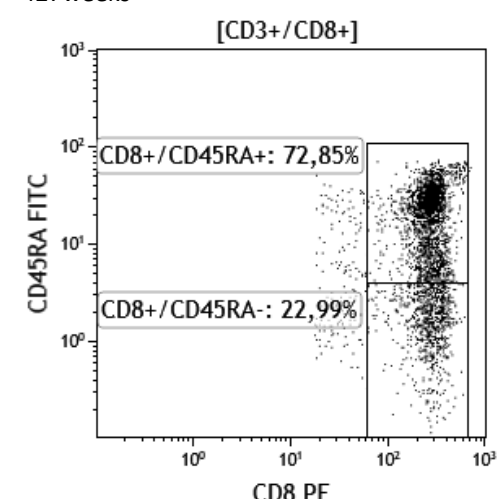

48-120 weeks

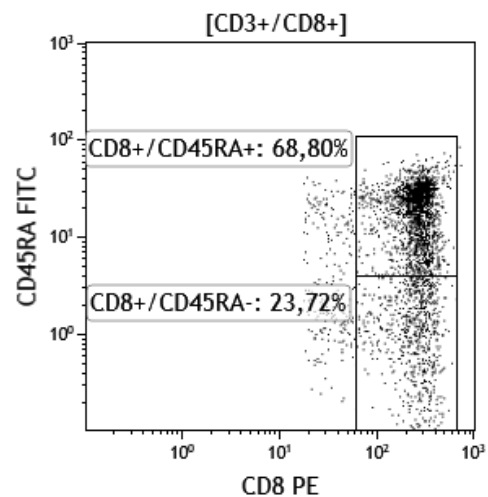

48-120 weeks

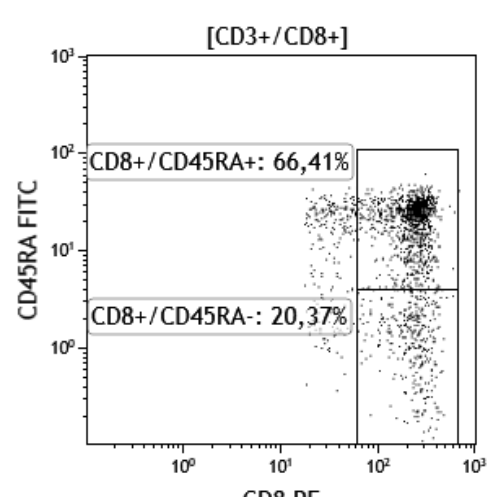

48-120 weeks

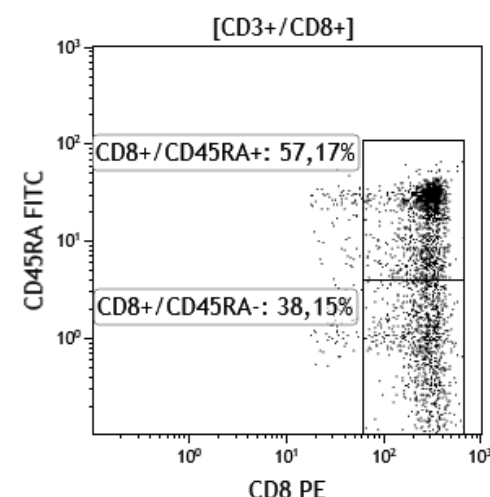

>120 weeks

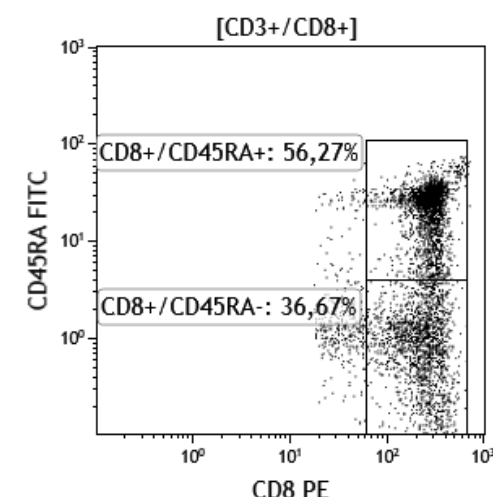

>120 weeks

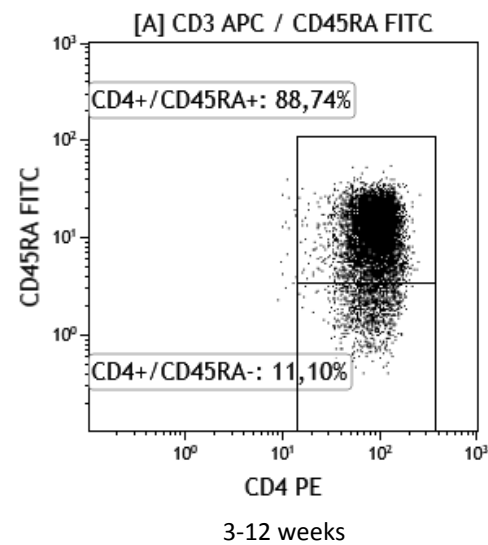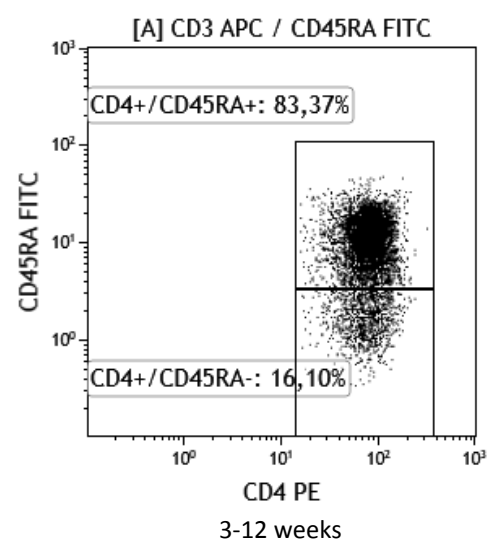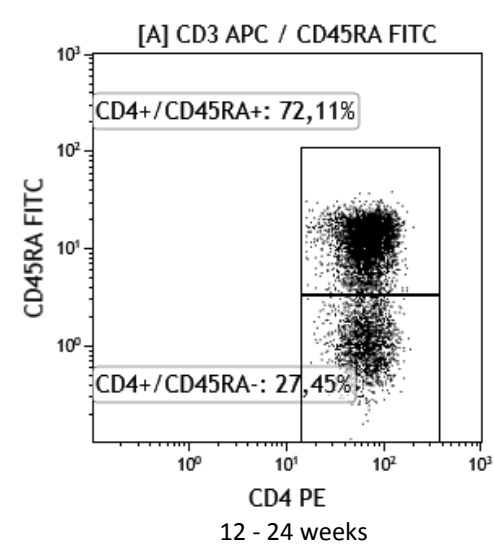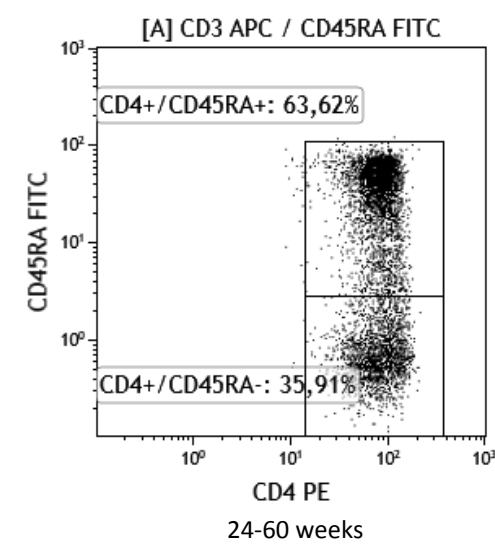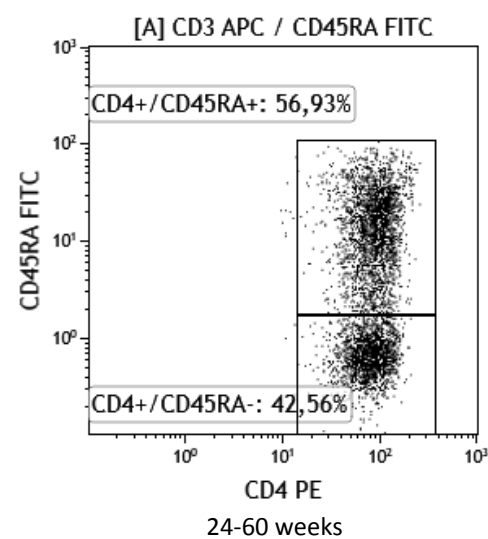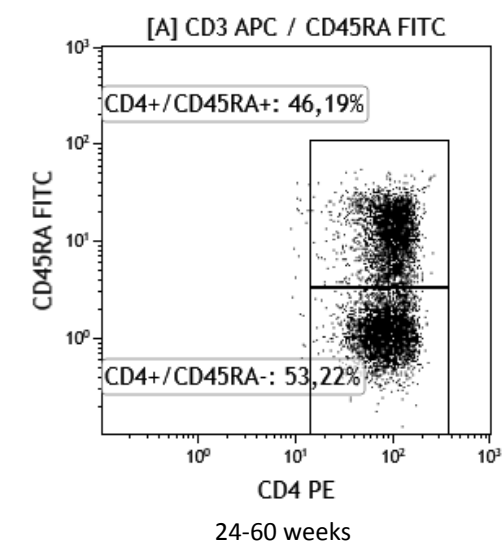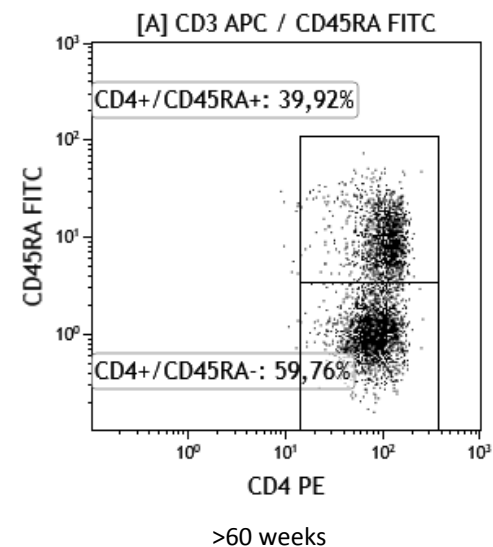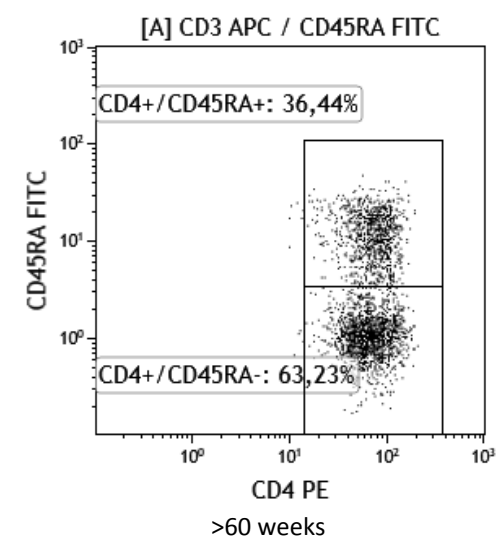

Supplement: Supplementary file 1 [file Data_Sheet_1.pdf]
