# Supplementary material for: Comparison of Lymphocyte Subset Populations in Children From South Africa, US and Europe
Source: Front Pediatr. 2020 Jul 23;8:406. doi: 10.3389/fped.2020.00406 (PMC7390891; doi:10.3389/fped.2020.00406)
Supplement: Supplementary file 2 [file Data_Sheet_2.pdf]

Supplement 1a

Gating strategy for the identification of CD45RA+ve/CD4+ve naïve, CD45RA+ve/CD8+ve naïve T-cell populations and the CD4+ve/HLA-DR+ve and the CD3+ve/CD8+ve/HLA-DR+ve activated T-cell populations.

A. The presence of the typical white blood cells found in peripheral blood samples were verified on forward versus side scatter dot plots.

B. CD3+ve T-cells were identified by placing a region around the CD3+ve lymphocytes on a CD3 versus side scatter dot plot.

C. CD3+ve/CD4+ve Helper T-cells and CD3+ve/CD8+ve Suppressor T-cells were further discriminated by using dot plots of CD3 versus CD4 and CD3 versus CD8.

D. The CD4+ve/CD27+ve/CD45RA+ve T-cells were subsequently isolated by gating on dot plots of CD27 versus CD45RA with the input gate from the previous dot plot of the CD3+ve/CD4+ve cells. CD8+ve/CD27+ve/CD45RA+ve T-cells were isolated by gating on dot plots CD27 versus CD45RA with the input gate from the previous dot plot of the CD3+ve/CD8+ve cells.

E. Further, dot plots of CD4 versus CD45RA and CD8 versus CD45RA were also created to compare the CD4/CD45RA expression to the expression of the CD4+ve/CD27+ve/CD45RA+ve T-cells and CD8/CD45RA expression to the CD8+ve/CD27+ve/CD45RA+ve T-cells as an internal control (A difference of <2% was acceptable for the final results analysis).

F. The CD3+ve/CD4+ve/HLA-DR +ve and CD3+ve/CD8+ve/HLA-DR +ve "activated" T-cell populations were identified using dot plots of CD4 versus HLA-DR and CD8 versus HLA-DR. The CD3+ve/CD4+ve/HLA-DR +ve expression was determined using an input gate from the CD3+ve/CD4+ve dot plot and the CD3+ve/CD8+ve/HLA-DR +ve expression determined using an input gate from the CD3+ve/CD8+ve dot plot with the region position determined using fluorescence minus one control (not shown in diagram).

The red arrows indicate the sequential gating strategy.

Supplement 1b

Examples of age related CD3+ve/CD8+ve versus CD45RA+ve dot plots from this study illustrating the observed trend where the CD8+ve/CD45RA+ve naïve suppressor T-cell populations were found to typically decrease as the age of the participants increased. The increasing CD8+ve/CD45RA-ve proportion of cells would be in keeping with the transition of CD3+/CD8+ naïve cells to CD3+/CD8+ memory cells.

Supplement 1c

Examples of age related CD3+ve/CD4+ve versus CD45RA+ve dot plots illustrating the observed trend where the CD4+ve/CD45RA+ve naïve helper T-cell populations were found to typically decrease as the age of the participants increased. The increasing CD4+ve/CD45RA-ve proportion of cells would be in keeping with the transition of CD3+/CD4+ naïve cells to CD3+/CD4+ memory cells.
